# Effects of the Molecular Structure of Starch in Foods on Human Health

**DOI:** 10.3390/foods12112263

**Published:** 2023-06-04

**Authors:** Jihui Zhu, Yeming Bai, Robert G. Gilbert

**Affiliations:** 1 Queensland Alliance for Agriculture and Food Innovation, Centre for Nutrition and Food Sciences, The University of Queensland, Brisbane, QLD 4072, Australia; jihui.zhu@uq.edu.au; 2 Jiangsu Key Laboratory of Crop Genomics and Molecular Breeding/Key Laboratory of Plant Functional Genomics of the Ministry of Education/Jiangsu Key Laboratory of Crop Genetics and Physiology, Agricultural College of Yangzhou University, Yangzhou 225009, China; yeming.bai@kuleuven.be; 3 Jiangsu Co-Innovation Center for Modern Production Technology of Grain Crops, Yangzhou University, Yangzhou 225009, China; 4 Laboratory of Food Chemistry and Biochemistry and Leuven Food Science and Nutrition Research Centre (LFoRCe), KU Leuven, B-3001 Leuven, Belgium; 5 School of Agriculture and Food Sciences, The University of Queensland, Brisbane, QLD 4072, Australia

**Keywords:** starch, fine structure, biosynthesis enzymes, models, digestibility, human health

## Abstract

Starch provides approximately half of humans’ food energy, and its structural features influence human health. The most important structural feature is the chain length distribution (CLD), which affects properties such as the digestibility of starch-containing foods. The rate of digestion of such foods has a strong correlation with the prevalence and treatment of diseases such as diabetes, cardiovascular disease and obesity. Starch CLDs can be divided into multiple regions of degrees of polymerization, wherein the CLD in a given region is predominantly, but not exclusively, formed by a particular set of starch biosynthesis enzymes: starch synthases, starch branching enzymes and debranching enzymes. Biosynthesis-based models have been developed relating the ratios of the various enzyme activities in each set to the CLD component produced by that set. Fitting the observed CLDs to these models yields a small number of biosynthesis-related parameters, which, taken together, describe the entire CLD. This review highlights how CLDs can be measured and how the model-based parameters obtained from fitting these distributions are related to the properties of starch-based foods significant for health, and it considers how this knowledge could be used to develop plant varieties to provide foods with improved properties.

## 1. Introduction

**Figure 1:**
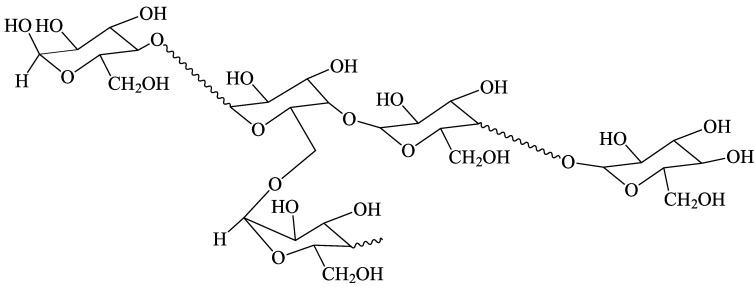
The chemical bonding in starch. The wavy lines represent many anhydroglucose units joined by (1→4)-α linkages.

A large proportion of meals in most societies contain rice, wheat, maize, taro or potato, and thus contain high proportions of starch [1,2,3,4]. This complex branched glucose polymer, with (1→4)-α linear links and (1→6)-α branch points (Figure 1), supplies the largest single component of food energy for most of the world’s population. Ordinary starch is almost entirely composed of two components: amylopectin and amylose. Amylopectin has a large number of short-chain branches and a high molecular weight, while amylose has a small number of long-chain branches and a lower molecular weight [5,6]. Starches from some plant varieties and from some mutants also contain intermediate structures [7].

The starch structure can be divided into different levels [8]. The lowest of these comprises individual chains of starch molecules. This is quantified by treating a starch sample with a debranching enzyme, breaking all the (1→6)-α branch points and measuring the number or weight distribution of the resulting linear chains, and thus yielding the chain length distribution (CLD): the number or weight distribution of chains (branches) as a function of the number of anhydroglucose monomer units in that chain.

The second level is the complex branched polymer comprising individual chains joined by (1→6)-α branch points. As stated, amylopectin and amylose have the same (1→4)-α and (1→6)-α bonding. Both have a broad distribution of molecular weights, with that of amylopectin typically approximately 10^7–8^, and amylose typically an order of magnitude less [5,6]. Amylopectin molecules are defined as having a large overall size and relatively short (relatively low degree of polymerization, DP) branches, while amylose molecules are defined as having a somewhat smaller size and relatively few branches, these having a significantly larger average DP than those in amylopectin.

The number-average branching fraction of rice amylose is up to 69%; it is 48% in maize and 44% in wheat. The number-average chain length of branched amylose molecules of rice covers the range ~230–370 (which is in the middle of the range of other crops), corresponding to branching fractions of 0.175 to 0.103 [9,10,11]. Short chains of amylose are usually defined as having DP < 100; while these are in the size range of the great majority of amylopectin chains, these short amylose chains have different CLDs from those of amylopectin [10]. Amylose is mainly found in the amorphous lamellae of the starch granule, although some portions of amylopectin chains could also be found there. When starch is heated in water, shorter amylose chains can leach out from the granular rings into the water because of the comparatively loose structure of the amorphous lamellae [12]. Analysis of the structures of granules shows that amylose is interspersed with portions of amylopectin molecules in lamellae [13]. In cereal starches, the amylose that has dissolved in the aqueous solution can be divided into lipid complex amylose and free amylose, of which only free amylose can interact with iodine and form iodine complexes, and thus be measured with the iodine test. The starch has to be defatted when measuring the total amylose content [9]. Note that the branching frequency, i.e., the probability of a monomer unit being branched, is the reciprocal of the average chain length.

A starch solution can be separated into amylopectin-dominated and amylose-dominated distributions, by separation by whole molecule size (such separation can also be achieved with butanol extraction, but this separation is less effective). The range of the CLDs in amylopectin is usually taken as chains with degrees of polymerization (abbreviation DP, symbol *X*) ≤ 100, or, to be more precise, at the DP where the weight distribution (usually obtained by size-exclusion chromatography, SEC) shows a distinct change, which is usually close to DP 100. Chains with DP less than this are usually assumed to belong to amylopectin, and those greater than this to amylose. It has been found that the CLDs of both the amylopectin and amylose components of starch—starch’s fine structure—are significantly correlated with various functional properties of starch, including digestibility and pasting properties [14,15,16,17,18,19,20,21,22,23,24,25].

A recent review [26] discussed the relationship between the overall starch structure and digestibility and thermal properties, and also summarized the digestion kinetics in rice. The present review complements this earlier review by considering more starch-containing species and focuses on the relations between CLDs and health-related properties. 

As will be seen, an understanding of these relations requires us to be able to represent the starch molecular structure in terms of a small number of parameters, which can subsequently be used in correlation analyses to discover biosynthesis–structure–property relations. In recent years, methods to conduct such a parameterization have been developed based on starch biosynthesis, with slightly different means of achieving this for amylopectin and for amylose. These take into account the activities of the three major enzymes in starch biosynthesis: isoforms of (1) starch synthase, SS; (2) starch branching enzymes, SBE; and (3) debranching enzymes, DBE. We also review methods to measure these CLDs. Knowledge of this fine molecular structure of starch, and the underlying biosynthetic mechanisms, helps the understanding of starch structure–property relationships. This can help breeders to target features of starch’s fine structure by breeding or genetic modification, so as to develop starch-based foods contributing to improved health and lifestyle outcomes.

## 2. Starch Functional Properties and Structural Features

### 2.1. Functional Properties of Starch-Based Foods and Human Health

When humans and other animals digest starch-containing foods, the starch is converted to glucose. The rate and location in the gastrointestinal tract of the digestion of starchy foods are significantly correlated with human health. Rapidly digested starch (RDS) leads to a spike in blood sugar, which can place a strain on the insulin system and, if this is chronic, can lead to diabetes. Slowly digested starch (SDS) reduces the glycemic load of a food product [27,28], which reduces the risk of type 2 diabetes, colon and breast cancers and cardiovascular diseases, and increases satiety [29]. Further, the consumption of foods with slow rates of starch digestion is helpful in controlling blood sugar for diabetics. Investigation of the postprandial physiological responses to the ingestion of RDS and SDS in healthy subjects and in subjects with type 2 diabetes has shown that insulin, blood glucose and non-esterified fatty acid concentrations are changed more significantly after the intake of RDS than by the intake of SDS [27,30]. It has been suggested [27] that the long-term intake of SDS is correlated with an improved metabolic profile and a reduction in the risk factors for metabolic syndrome. Epidemiological studies show that diabetes is correlated with the glucose intake from foods, and it is suggested that the reduction of postprandial glucose peaks in the bloodstream is beneficial in the management of diabetes. The intake of slowly digestible starch can lower postprandial glucose peaks [27,30,31,32]. It has also been shown that carbohydrate metabolism in humans is improved (by which is meant a slower digestion rate) by the intake of foods containing significant amounts of SDS, and this also reduces the insulin requirements of insulin-treated type 2 diabetic patients [33]. Moreover, the resistant starch (RS) that reaches the large intestine is a substrate for microbial fermentation; hydrogen, carbon dioxide, methane and short-chain fatty acids are end-products from this fermentation, which can improve the health of the intestinal system [34] and attenuate postprandial glucose and insulin responses, which are beneficial for human health [35,36].

The digestion rate of starch also has correlations with human mental health. For example, it has been reported that the level of blood glucose can influence mental performance, especially for demanding tasks such as memory, and that a breakfast enriched in SDS can reduce the possibility of a decline in performance later in the morning [37]. It has been postulated that hunger is triggered by low blood glucose concentrations, while high blood glucose levels signal satiety [38,39].

The digestibility of starch in food can depend on the method used in processing this food. It has been reported [40] that the digestibility of raw starch and starch cooked in excess water and in limited water is different, where starch cooked in excess water shows the fastest digestibility. Normally, starchy foods, such as rice, maize and wheat, are heat-treated before consumption, making the starch pasting and gelatinizing properties important. Measuring the pasting profile usually involves heating samples slowly in water to a temperature higher than 80 °C, followed by cooling, while simultaneously measuring the viscosity, which can be performed with a device such as a rapid visco-analyzer (RVA). The viscosity of the suspension of the sample in water as a function of time/temperature is called the pasting (or RVA) profile [41]. The various features of the pasting profile are the peak viscosity (PV), hot paste viscosity (HPV), cool paste viscosity (CPV), pasting temperature (PaT) and peak time (PeT). The breakdown viscosity (BDV), setback viscosity (SBV) and consistency viscosity (CSV) can be calculated from these parameters; BDV is the difference between PV and HPV, SBV is the difference between CPV and PV, and CSV is the sum of BDV and SBV [42]. Sensory properties are significantly correlated with pasting properties [23].

### 2.2. Distributions

The structure of a glucose polymer can be quantified in terms of various distribution functions. As an example, for a whole starch molecule, one such distribution is the number distribution of molecules as a function of their size. Another common distribution is the weight distribution: the weight of molecules as a function of their size, *w*(log*R*_h_).

### 2.3. The Size of a Starch Molecule in Solution

Consider a homopolymer molecule in a solution comprising atoms or monomer units of mass *m* located at distances *r_i_* from the center of mass. One definition of size for any polymer molecule in a solution at any instant is the root-mean-square radius of gyration, defined as the square root of the mass average of *r_i_* (Definition 1.9 in the 1988 edition of the IUPAC “Purple Book” of polymer terminology [43]). In a solution, the position of each atom or monomer unit in an individual polymer molecule is constantly changing, and the “size” therefore also fluctuates. As the individual units are constantly fluctuating, one has

$$<s^{2}{>}^{1/2}=<\sum_{i}{r_{i}}^{2}{>}^{1/2}$$

where <*s*^2^>^1/2^ is the root-mean-square radius of gyration, the sum is over all the atoms or monomer units in the polymer and the angular brackets denote a long-time average. For a polymer molecule in a solution, this quantity cannot be directly observed, and a size measurement requires an appropriate experimental technique and accompanying theory. Multiple-angle laser light scattering (MALLS) gives the average radius of gyration, as above; it also gives the weight-average molecular weight.

Another commonly used size parameter for polymers is the SEC hydrodynamic radius *R*_h_ (or hydrodynamic volume *V*_h_ = 4/3 π *R*_h_^3^). The IUPAC definition of the hydrodynamic radius specifically depends on the method of measurement [43] (Definition 3.2.2 in the IUPAC “Purple Book” [43]). The definition of the SEC hydrodynamic radius starts with the “universal calibration” assumption [44]—the notion that polymer molecules separate in SEC solely by their sizes (defined as *V*_h_ or *R*_h_) in the solvent, independent of their structure and composition. It is noted that this assumption has only been subjected to limited testing [44].

Universal calibration is implemented using approximately monodisperse samples of standards (pullulan being suitable for starch) with a suitable range of molecular weights, and hence of values of *R*_h_, these having been measured independently, e.g., by MALLS, using the same solvent and SEC set-up (columns, flow rate, temperature, etc.) as those for the analyte. With the universal calibration assumption, one can then plot the elution volume as a function of *R*_h_, giving a calibration curve. Note that this varies with the column(s) (including column age), temperature, solvent and other conditions, and even can show significant day-to-day variability; calibration must be carried out daily.

The preceding discussion is applicable to any polymer, branched or unbranched, of any composition. For a linear homopolymer, there is a one-to-one relation between the size of a molecule and its molecular weight, or its degree of polymerization *X*; the weight distribution in terms of size can thus be converted into the weight distribution in terms of the degree of polymerization, *w*(log*X*). For debranched starch, which is a linear polymer, one has *M* = *M*_0_*X* + 18, where *M*_0_ = 162.2 is the molecular weight of the anhydroglucose monomer unit and 18 is that of the additional water in the end groups.

There is, however, no such unique relation between size and molecular weight with a (complex) branched polymer such as starch; complex branched polymers with different molecular weights and different structures can have the same hydrodynamic size. The best that can then be obtained with light scattering detection is the relative weight, and hence concentration, of the sample in that elution slice as a function of *R*_h_, *w*(log *R*_h_) and the weight-average molecular weight, $\bar{M_{w}}$ (either averaged over *R*_h_ in the whole sample, or as a function of *R*_h_ for size-separated samples).

The CLD is a significant determinant of starch’s digestibility and pasting properties [14,15,16,17,18,19,20,21]. It is obtained by measuring the weight or number distribution as a function of the DP of the linear glucans obtained after the quantitative enzymatic debranching of a starch sample, using a weight- or number-sensitive detector as appropriate. This involves cutting each (1→6)-α link, using an isoamylase debranching enzyme, to produce linear glucan chains. The CLD can equally well be expressed as the number CLD, *N*_de_(*X*) (the number of chains after debranching with degree of polymerization *X*), or the weight CLD, *w*_de_(*X*), or often simply *w*(log*X*). For a linear homopolymer, the two distributions are related by [45]

*w*(log*X*) = *X*^2^ *N*_de_(*X*).

**Figure 2:**
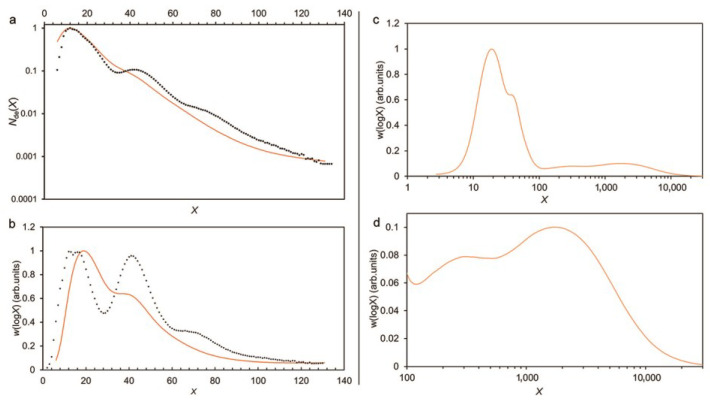
Typical amylose and amylopectin CLDs, in terms of the degree of polymerization of the (debranched) chain, obtained by Dr. Kai Wang and Dr. Haiteng Li in the authors’ laboratory. Left panel: amylopectin for a rice sample, obtained by both FACE (points) and SEC (lines). FACE data shown as points, because FACE resolves individual degrees of polymerization. Left panel shows (**a**) number distribution *N*_de_(*X*) and (**b**) SEC weight distribution *w*(log*X*) = *X*^2^ *N*_de_(*X*); (**b**) the SEC weight distribution of the same sample, as a line, showing the band broadening that occurs in SEC. Right panel: (**c**,**d**) whole weight CLD for a rice sample.

Examples are given in Figure 2, which shows typical amylopectin and amylose CLDs. 

### 2.4. Measuring Distributions Related to Starch Structure

There are a number of techniques to measure the starch structure, for both the whole molecule and the individual chains (branches) obtained following the breakage of each (1→6)-α linkage (branch point) with a debranching enzyme. The distribution of the individual chains thus obtained is the CLD.

One method to measure the CLD is fluorophore-assisted carbohydrate electrophoresis (FACE) [46]. To prepare the samples for measurement, debranched chains are labeled with a fluorophore such as 8-amino-1,3,6-pyrene trisulfonic acid, and then are subjected to electrophoresis with a fluorescence detector. Individual degrees of polymerization can be, in favorable cases [47], baseline-separated up to DP ~ 180, which gives accurate results for all but extra-long amylopectin chains; it also gives results for the shortest chains of amylose.

A method to measure both the CLD and the size distribution of whole molecules is size-exclusion chromatography (SEC, a type of gel permeation chromatography, GPC), which is suitable to measure the entire DP range of both debranched amylopectin and debranched amylose [48,49,50]. It is essential to be aware that SEC separation is not based on molecular weight but molecular size, which, as noted above, is defined as the SEC hydrodynamic radius.

SEC suffers from band broadening (unlike FACE). This is an unavoidable problem for SEC [51], whereby a given elution slice contains “leaks” from nearby regions of *R*h during the passage of the polymer solution through the column. When using SEC on whole amylopectin molecules, band broadening means that features with close but different molecular size ranges may blend into what seems to be a single feature (Figure 2). SEC is also subjected to uncertainties in the calibration assumptions and parameter values required to convert the SEC elution time into the DP [50,52,53,54]. 

There are various types of detectors for SEC. The differential refractive index (DRI) is the difference in the refractive indices of the polymer solution and pure solvent and is proportional to the mass of the polymer in the solution; a DRI detector gives the SEC weight distribution in terms of the DP, *w*(log*X*), or in terms of the hydrodynamic radius, *w*(log *R*_h_). The angular dependence of the intensity of MALLS is sensitive to the weight-average molecular weight, $\bar{M_{w}}$, and the average radius of gyration, *R*_g_, of the sample in the elution slice. If the Mark–Houwink parameters of either sample or standard are unavailable, then this will give the molecular size distribution relative to the standard, but not the absolute value. A viscometric detector is sensitive to the intrinsic viscosity and thus (with knowledge of the Mark–Houwink parameters relating the viscosity and molecular weight of a linear polymer) the number-average molecular weight, if the sample is unbranched.

SEC has some disadvantages, especially with regard to the assumptions needed for calibration. As mentioned above, SEC separates particles by hydrodynamic radius [44]. This separation parameter is a complex quantity, and the only study of this at present [44] suggests that it is proportional to the product of the weight-average intrinsic viscosity and the number-average molecular weight. A differential refractive index (DRI) detector gives the weight of the polymer in the elution slice as a function of hydrodynamic volume: *w*(log *R*_h_). The independent variable is expressed logarithmically, as SEC elutes approximately (but not exactly) linearly with the size of an unbranched polymer over a moderate range, where *w*(log *R*_h_) or *w*(log*X*) is approximately proportional to log *R*_h_ or log*X*, respectively. A MALLS detector gives the weight-average molecular weight and the average radius of gyration of the polymers in an eluent slice.

When a MALLS detector is unavailable, the conversion from the SEC elution time to DP can be achieved by calibration with relatively monodisperse standards with known *R*_h_, measured in the same solvent, temperature and SEC set-up and assuming the validity of the Mark–Houwink relation over the DP range of interest [50] and the availability of the Mark–Houwink parameters for the polymer and solvent. The Mark–Houwink relation is an assumption about the connection between the solution viscosity and the molecular weight of a linear polymer.

Another method to measure the CLD is high-performance anion-exchange chromatography at a high pH with pulsed amperometric detection (HPAEC), which can be used up to DP ~ 60. However, this method suffers from mass bias [55,56], which is difficult to correct, leading to only semi-quantitative results. It is, however, the only method that can be used if more than one polymer composition is present.

### 2.5. Interpreting Experimental CLDs

A common method to interpret size distribution data involves calculating the average degree of polymerization ($\bar{DP}$ or $\bar{X}$) of chains within a specific range. However, this is influenced by the arbitrary selection of the ranges, and there is no standard for the choices. A preferred approach is to fit the obtained data with biosynthesis-based models for both amylopectin and amylose [8,57,58], as discussed in Section 3. 

### 2.6. Two-Dimensional Distributions

**Figure 3:**
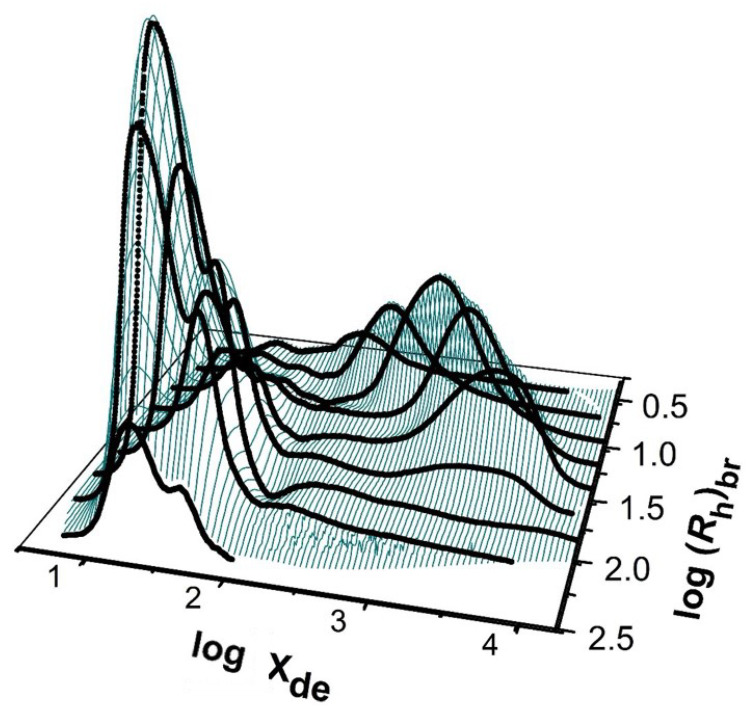
Two-dimensional structural distribution of a normal maize starch. The Z axis (arbitrary units) is the relative weight of molecules in the elution slice; the Y axis is the total molecular size (as the hydrodynamic radius of the fully branched molecule); and the X axis is the degree of polymerization of an individual branch (following debranching). Replotted from data in [7]. The feature toward the left is the amylopectin “mountain”, while that toward the right is the amylose “mountain”.

Defining amylopectin molecules as having a large total molecular size and short chains, and amylose molecules as having a smaller total size and long chains, can lead to ambiguity, as is the case with high-amylose rices (e.g., [59]). This problem disappears if one has two-dimensional distributions [60,61], with one dimension being the chain length or DP *X* and the other being the total molecular size, *R*h, of the whole (fully branched) polymer. For ordinary starch, in a 2D distribution, one expects to see two “mountains”: one for long chains and a small total molecular size (amylose) and the other for short chains and a large total molecular size (amylopectin). Such distributions are very laborious to obtain experimentally [62]. First, a large (“preparative”) SEC column is used with fraction collection to prepare a series of samples that are relatively monodisperse in total molecular size (*R*_h_) and are sufficient in amount to be subsequently subjected to enzymatic debranching. This is then followed by SEC analysis of the resulting debranched chains. Such a procedure is termed SEC × SEC separation. An example of this for ordinary starch is shown in Figure 3; special starches such as high-amylose varieties are rather different [7].

### 2.7. Distributions of Whole Starch Molecules

The weight distributions of whole starch molecules have not been extensively studied. This can be ascribed to two problems.

The first problem is that the commonest method for obtaining such distributions, SEC, results in shear scission with the larger starch molecules on the SEC columns and tubing, and extensive data together with dimensionless analysis [51] show that this is unavoidable. Moreover, not only does shear scission vitiate the apparent size distributions of the larger starch molecules, but it also vitiates the data for smaller sizes, because of the contamination of smaller sizes with fragments from shear scission. This problem cannot be avoided by simply reducing the flow rate through the columns, because this results in poorer size separation.

One way to overcome this would be to use a size separation technique with lower shear than that in SEC. One such technique is asymmetric-flow field-flow fractionation, AF^4^ [63]. However, there do not appear to be any published studies of the size distributions of whole starch molecules using this technique, where checks have been carried out to ensure that the following two conditions are met: (a) the starch must be completely dissolved without molecular degradation—this requires dimethyl sulfoxide in the dissolution procedure, but this solvent degrades the membrane used in AF^4^; (b) the whole size range of native starch is covered. Some excellent studies on smaller starch-like polymers have appeared, e.g., [64,65,66,67], but none on the whole size distribution of native starch. This is a potentially fruitful area in which to gain knowledge, for example, on the biosynthetic processes controlling the growth of whole starch molecules.

## 3. Biosynthesis, Measurement and Fitting of the CLD

### 3.1. Enzymes Controlling the CLD

**Figure 4:**
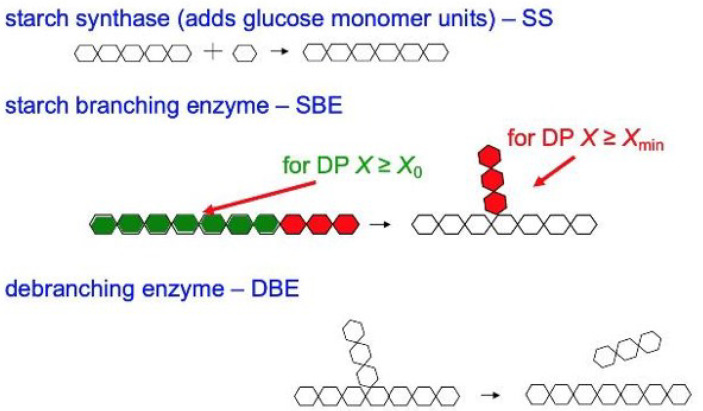
The main steps in the biosynthesis of starch. For amylopectin, SS is starch synthase, while, for amylose, it is granule-bound starch synthase.

The biosynthesis of starch is shown in Figure 4. Starch is mainly biosynthesized by five enzymes: ADP-glucose pyrophosphorylase (AGPase), starch synthase (SS), starch branching enzymes (SBE) granule-bound starch synthase I (GBSSI, pronounced “GBSS one”) and starch debranching enzymes (DBE) [68,69,70,71,72,73,74,75,76]; each one of these has a number of isoforms. SS, SBE and DBE are the major enzymes for the biosynthesis of amylopectin, while amylose is mainly controlled by GBSSI, SBE and DBE [77,78,79,80,81]. DBE can hydrolyze the (1→6)-α glucosidic linkages of polyglucans directly [72,76], while SBE cuts an (1→4)-α link and then adds the short chain to the parent chain or to another chain to form an (1→6)-α linkage branch point [74,75]. Isoamylase and pullulanase are the two isoforms of DBE; isoamylase is believed to remove improperly spaced branches and/or edit over-long chains of amylopectin [76]. The isoforms of SBE are involved in the synthesis of both amylopectin and amylose. Importantly, SBE only operates on a chain longer than a certain length, with DP > *X*_min_~6 [75]. An amylose extender (*ae*) mutation of SBEIIb has been found to suppress SBE and can significantly increase the amount of amylose chains [74,76,82,83]. However, the effects of the same type of isoform in different crops might be slightly different.

Each CLD can be seen as the sum of contributions from “enzyme sets” [84], which comprise one (and sometimes more) isoform of each of an SS (GBSS for amylose), SBE and DBE. The biosynthesis-based parameterization of a CLD is implemented by fitting data with biosynthesis-based models for both amylopectin and amylose. The models assume that the dominant, but by no means only, contribution to the CLD in a given DP range is from a particular enzyme set. The ranges of chains synthesized by these sets overlap, although a feature such as a maximum in the CLD is dominated by the contribution from a single set. 

In addition to isoforms of SS, SBE and DBE, another enzyme involved in starch biosynthesis is AGPase, which catalyzes and produces ADP-glucose. As the first step in starch biosynthesis, ADP-glucose is elongated by soluble starch synthases (SSs) through (1→4)-α linkages [68,69]. There are a number of SS isoforms in rice, especially in the endosperm [68]: SSI, SSII-1, SSII-2, SSII-3, SSIII-1, SSIII-2, SSIV-1 and SSIV-2 [71,72]. SSI is a major isoform of SS and preferentially works on the shortest amylopectin chains as substrates. Short amylopectin chains are extended by SSI up to a critical length (DP > 12) [72]. SSII has three isoforms, one of which is SSII-3 (encoded by the *Alk* gene), which is significantly correlated to the gelatinization temperature of rice grains, and so affects rice cooking quality [72,85]. The behavior of SSII-3 varies in different rice sub-populations; it can elongate short chains of DP < 11 to form chains of DP 13–25. While it has no effect on the proportion of DP < 29 chains in certain transgenic Indica plants, this shift in chain length was not observed with *japonica* SSII-3 [86,87,88,89,90]. In rice SSIII-2 mutants, it was found that the chains of DP 6–8, DP 16–20 and DP < 30 were reduced, whereas the chains of DP 9–15 and 22–29 were increased [70,71]. Studies of SSIIIa mutant rices indicate that, in the rice endosperm, this enzyme controls the biosynthesis of DP > 30 amylopectin chains from intermediate chains [70]. Although SSIV has been detected during grain filling, it has not yet been widely studied; work on *Arabidopsis* [72] shows that SSIV mutants display little or no shift in the amylose/amylopectin ratio and chain length distributions. However, SSIV Arabidopsis mutants show a striking reduction in the number of starch granules and an increase in the granule size compared to the wild type [68,69,70,71,72,73].

GBSS both elongates amylose chains and increases the number of extra-long chains (ELCs) of amylopectin, while SS I–IV are believed to mainly act in amylopectin synthesis [72,77,91,92]. Usually, GBSS has two isoforms, but only one of these, GBSSI, acts on the seed endosperm [93]. GBSSI is encoded by the *Waxy* (*Wx*) gene, which mainly controls amylose biosynthesis [86,93,94,95].

It was first reported that GBSSI uses non-physiological concentrations of UDP-glucose, but ADP-glucose was subsequently discovered to be the preferred substrate [96]. GBSSI activity has been found to be confined to a core within the starch granule where amylose is synthesized. The starch in this core, the size of which was found to be dependent on the amount of GBSSI protein present, is indistinguishable from normal wild-type starch and contains the same amylose:amylopectin ratio. It was reported that GBSSI-catalyzed amylose synthesis requires the presence of small malto-oligosaccharides, and it was suggested that these could trigger amylose synthesis [96,97]. It was also found that GBSSI could participate as a minor component of amylopectin synthesis when there is a lack of malto-oligosaccharides, and that the maximum size of the soluble malto-oligosaccharide that can interact with GBSSI depends on the porosity of the starch [96,97]. These studies indicated that long-chain amylose synthesis precedes that of short-chain amylose, which led to the hypothesis that GBSSI was continuously using amylopectin as a primer and extending a long outer chain. This suggests that a high-molar-mass polysaccharide is being used by GBSSI in the granule to extend the amylose chains. However, no evidence has yet been found to prove that the GBSSI could directly use amylopectin to extend a longer chain; there is also the possibility that the short chains all result from branching and then are used by GBSSI to extend the amylose chains.

It is found that some enzymes work on more than one DP range. Some examples of this are as follows: (1) SBE takes part in amylose synthesis [98,99], as well as that of amylopectin; (2) it is found that mutations of SSIIa and SBEIIa can increase the amylose content [100]; (3) different enzyme sets of SBE and SS in amylopectin are also involved in the synthesis of rice amylose—GBSS, SBE and SS act together to catalyze the biosynthesis of amylose and amylopectin [101]; (4) SBEIIa modifies shorter chains (degree of polymerization DP ≲ 12) in amylopectin and longer chains in amylose (with a CLD peak at DP ~ 3000), while SBEIIb acts on longer branches (DP ≲ 32) in amylopectin [102]. Presently, researchers have found a total of 12 isoforms of the three major starch biosynthesis enzymes (SS, SBE and GBSS): SSI, SSII-1, SSII-2, SSII-3, SSIII-1, SSIII-2, SSIV-1, SSIV-2, SBEI, SBEIIa, SBEIIb and GBSSI [70,71,72,74,75]; note that names such as SS can be used to refer to different enzyme types in different species. Based on the relationships between the mutations of these 12 isoforms and their starch structural features, Zhu et al. [103] found that these isoforms could belong to different enzyme sets and affect the biosynthesis of both amylopectin and amylose. It is reported that these isoforms can belong to five different enzyme sets, denoted as amylopectin enzyme set i-iii and amylose enzyme set i-ii [101,103]. For instance, SS (SSI, SSII-2, SSII-3, SSIII-2, SSIV-1 and SSIV-2), SBEI and GBSSI can form amylopectin enzyme set i; amylopectin enzyme set ii is formed by SSII-1, SSII-2, SSII-3, SSIII-1, SSIII-2, SSIV-1 and GBSSI; the components of some amylopectin enzyme sets are relatively simple, comprising SSII-3 and GBSSI; furthermore, SS (SSI, SSII-3, SSIII-1 and SSIII-2), SBEII and GBSSI can form amylose enzyme set i, and SS (SSI, SSIII-1 and SSIII-2), SBE (SBEI and SBEII) and GBSSI form amylose enzyme set ii [8,70,72,76,83,88,89,90,91,92,98,99,101,103,104,105,106,107,108,109,110,111]. Table 1 shows which isoforms belong to which enzyme sets and the effects of the enzyme sets on the structural features of starch.

### 3.2. Fitting Observed CLDs to Biosynthesis-Based Models

Fitting the observed CLDs to biosynthesis-based models is useful because the parameters resulting from the fitting, which together enable the whole CLD to be reconstructed, are biologically based. This should replace what is often performed at present, which involves the relative number of chains in the CLD in empirically chosen DP ranges. An empirical DP division is problematic because the inferences might change if the choice of region was to be changed [8].

**Figure 5:**
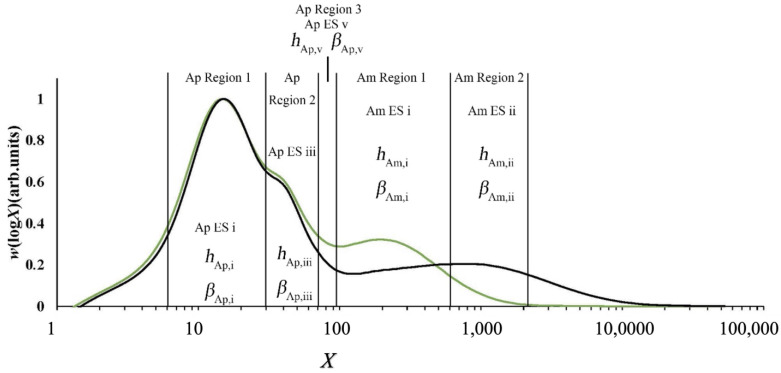
SEC weight distribution *w*(log*X*), showing the fitting regions and corresponding parameters of two typical rice samples, CH1069 and G259. *X*, degree of polymerization; Ap, amylopectin; Am, amylose; ES, enzyme set; redrawn from data in [103]. Green: G259; black, CH1099.

This problem can be avoided because of the development of biosynthesis-based models, which include the assumption that the DP in a given range of DP is dominated by an enzyme set, but there are contributions from other enzyme sets as well, as shown in Figure 5 [103]. There are slightly different treatments for amylopectin and amylose.

The amylopectin model developed by Wu et al. [57] assumes that amylopectin biosynthesis is controlled by different independent enzyme sets in which different isoforms of SS, SBE and DBE are involved. For both amylopectin and amylose, the requirement that the CLDs be in a steady state (i.e., do not change in time) reduces the model to only two parameters for each enzyme set: the ratio of the activities *β_i_* of SBE and SS for each enzyme set *i*, and the relative propagation rates *h_i_* of SS in these sets [57]. Briefly, *h_i_* represents the relative number of branches in given region *i*, and *β_i_* reflects the number of shorter chains in region *i*. The theory gives a means to calculate the contribution to the overall CLD from each enzyme set, and the fitting starts by assuming only a single enzyme set in a chosen DP range. The first sets of values of the parameters *β_i_* and *h_i_* thus obtained are then refined in the next step of the data fitting, which takes all enzyme sets into account and applies a global least-squares fit to the entire DP range.

For amylose, the model developed by Nada et al. [58] also involves two fitting parameters, *β* and *h*, which are analogous to those for amylopectin [8]. Similar to the model of amylopectin, the amylose model assumes that amylose CLDs are controlled by different individual enzyme sets, including GBSS (for chain growth, possibly with other SSs) and SBEs (for chain stoppage) [58].

For amylopectin, data fitting yields *β*_Ap, *j*_, the ratio of activities of SS and SBE in enzyme set *j* (*j* = i, iii, v) and the number amount of the overall CLDs produced by this set to that produced by that *j*: *h*_Ap, *j*_. DBE is also involved, but the mathematical development using the requirement for steady state shows that its activity is determined by those of SS and SBE. For amylose, the amylose fitting parameters, *β*_Am, *j*_ (*j* = i, ii) and *h*_Am, *j*_ (*j* = i, ii), have the same meaning as those for amylopectin [57,58].

The models used to interpret these data were derived by extending the theory of the molecular weight distributions of linear polymers developed originally for free radical polymerization [45]. The code to fit experimental CLDs to these models is publicly available without cost [58,112]. The mathematical expressions for the CLDs satisfactorily reproduce the experimental data using the fitted values of *β_i_* and *h_i_* for each enzyme set. The most important use of the model fitting is that it reduces an experimental CLD to a small number of biosynthesis-based parameters, and that these parameters, together encapsulating the CLD over the entire range of starch chain lengths, may then be used to seek correlations between the structure and properties of interest. With each such correlation, it is essential to distinguish statistical coincidence from causal relations; for this purpose, one must determine whether a physically reasonable mechanism can be postulated that is consistent with each correlation—for example, that the dominant events in chain growth and stoppage are growth (propagation) by a starch synthase and stoppage by a debranching enzyme. This process can reveal mechanisms in starch biosynthesis.

## 4. Relationships between Starch Fine Structure and Health-Related Properties

### 4.1. Relationship between Molecular Fine Structure of Starch and Digestibility

Starch’s fine structure is an important factor that can affect digestibility and other functional properties, some examples of which are reviewed here. Only studies on cooked starches are considered, as humans rarely eat raw starch.

1. A number of studies have reported that the DP 6–12 region of amylopectin affects the amount of RDS and SDS. It was reported that a lower proportion of short A chains (DP 6–12) was correlated with lower RDS and could contribute to a higher SDS in indica long-grain rice [113]. It was found that the proportion of short chains of cooked maize starch, especially short A chains (DP < 13) in amylopectin, was negatively correlated with the rate of enzyme digestion because of the short chain length and higher branch density [114].

2. The DP 13~100 region of amylopectin can also be significantly correlated with digestibility. It was reported that a higher proportion of long amylopectin chains (DP ~ 37) in *indica* long-grain rice had a lower RDS [113]. In addition, it was also reported that a high proportion of long amylopectin B chains (DP > 40) could affect SDS retrogradation on cooling, which contributes to slow digestion. This was speculated to arise from an anchoring effect of crystallites that were formed by some short B chains (mainly B1 chains, DP 13–30) with the longer chains (B2–B4, DP 30–69) and outer A chains (DP 9–13) in maize [115].

Amylopectin, with a high branching density, short chains and shortened terminal nonreducing ends, is more slowly digested in cooked starch in maize: more and shorter short chains are slow to digest [115,116,117]. This might be because α-amylase prefers to cleave (1→4)-α glycosidic linkages, while the rate of cleavage of (1→6)-α linkages by amyloglucosidase is much slower than the cleavage of (1→4)-α linkages; however, for native granular starch, a certain pattern of CLDs is closely related to the high crystallinity of granules with a compact structure or smooth surface. The rigid granular structure of starch is not favorable for amylases to bind and hydrolyze. Pullulan, from fungus and potato, which has maltotetraose and maltotriose as basic structural units and has shorter linear chains, is slowly digestible [117,118]. 

The chain length distribution of amylose also significantly affects digestibility, as illustrated in the following examples.

1. In the DP 100–500 region of amylose, i.e., short to medium chains, it was found that the in vitro digestibility of native and cooked rice starches was influenced by both the degree of branching and amylose content [119,120,121]. Short amylose chains formed by debranching waxy starches can form double helices that aggregate into ordered crystalline arrays during cooling, and they are slowly digested. On the other hand, longer amylose chains (intermediate amylose chains, DP ~ 500–1000) in non-waxy native and cooked rice starch can prevent aggregation and thereby form a cross-linked network during cooling, which can slow the rate of digestion [119,120,121]. Furthermore, in addition to the amylose content, it has been found that amylose’s fine molecular structure is a major factor controlling the digestibility of cooked and retrograded rice starch, and that the short–medium chains of amylose have the greatest effect on digestibility [122].

2. The DP 1000~1500 region of amylose also has an effect. The long chains of amylose are defined as DP 500~1500. At present, the effects of amylose’s molecular fine structure, and the interactions with amylopectin molecules on starch digestibility, have been less studied. A study of cooked and retrograded rice starch reported that amylose long chains correlated with digestibility in rice, but the correlation was not as significant as that of amylose short–medium chains [122].

The relationships between starch structural features and digestibility are shown in Table 2.

### 4.2. Relationship between Starch Molecular Fine Structure and RVA Profile

**Figure 6:**
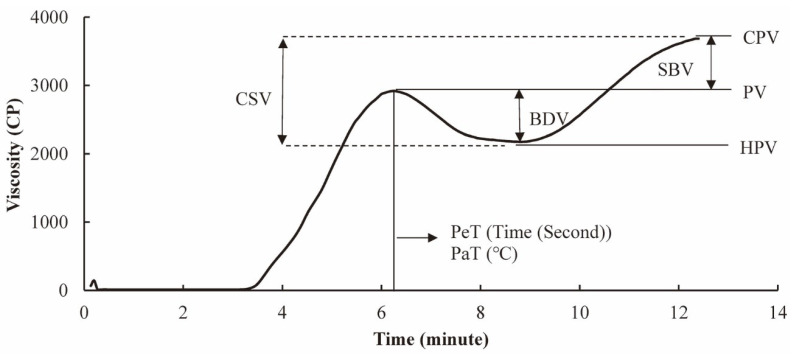
A typical RVA profile of rice with the explanation of different parameters. Notes: PV, peak viscosity; HPV, hot paste viscosity; CPV, cool paste viscosity; BDV, breakdown viscosity; SBV, setback viscosity; CSV, consistency viscosity; PaT, pasting temperature; PeT, peak time.

The rapid visco-analyzer (RVA) profile is affected by starch’s fine structural features, such as the chain length distributions of amylopectin and amylose. Both affect the rheology of suspensions of rice flour. A typical rice RVA with the explanation of different parameters has been shown in Figure 6. The effect is higher when the grain has more medium-chain-length amylose molecules and longer amylopectin branches [41]. The increase in viscosity seen when heating starch in water using an RVA is affected by the swollen granules, while the breakdown in viscosity is mainly caused by the breakdown of gelatinized starch granules [123]. The parameters that fit the measured heat flow and viscosity for this breakdown are found to be correlated with eating and cooking qualities [123,124,125]. The proportion of long chains of amylopectin is negatively correlated with breakdown; on the other hand, the proportion of short chains of amylopectin is positively correlated with breakdown. The relationships between the starch structural features and the RVA profile are shown in Table 2.

## 5. Conclusions and Future Perspectives

Starch’s molecular fine structure is a determinant of its functional properties. Starch’s structural features can be interpreted and fitted using biosynthesis-based mathematical models [57,58]. A large number of studies have explored the relationships between starch’s structural features and functional properties, including digestibility and pasting properties, as seen in Table 2. For example, one study [128] indicated that a high-amylose RS diet prevented the fragility of the liver glycogen α particles of diabetic mice, this fragility having been found to be a characteristic of diabetes; this proves that starch’s fine structure can affect health-related indicators in vivo. However, more information is needed on the detailed effects of starch’s fine structure on health. Further studies could be focused on the correlation between amylopectin intermediate and long chains and digestibility, to obtain a method to improve the health-related properties of starch-based foods, and on correlations with the parameterization of the amylose CLD [57,58] and appropriate properties. It is well known that the amylose content affects the digestibility and other properties, but the effects of the CLD of amylose have not been fully studied. In addition, more work needs to be performed on the relationship between starch’s fine structure and its pasting properties.

This review addresses the relationship between starch’s fine structure and its functional properties, indicating the potential of modifying starch’s structural features to improve the health benefits of starchy foods. This can help breeders to target starch’s fine structure using conventional and GM methods [129], to adjust the digestibility and pasting properties and to further develop starch-based foods with desirable qualities, leading to improved human health. 

## Figures and Tables

**Table 1:** Steps controlled by the various isoforms [103]. (Ap, amylopectin; Am, amylose).

Isoform	Enzyme Set	CLD Control	DP Range
SSI	Ap enzyme set i, Am enzyme set i, Am enzyme set ii	Ap short chains, Am short and intermediate–long chains	6–24, 100–500; 500–1500
SSII-1	Ap enzyme set iii	Ap intermediate chains	28–58
SSII-2	Ap enzyme set i, Ap enzyme set iii	Ap short and intermediate chains	6–24; 28–58
SSII-3	Ap enzyme set i, Ap enzyme set iii, Ap enzyme set v, Am enzyme set i	Ap short, intermediate and long chains, Am short chains	6–24; 28–58; 100–500
SSIII-1	Ap enzyme set iii, Am enzyme set i, Am enzyme set ii	Ap intermediate chains, Am short and intermediate–long chains	28–58; 100–500; 500–1500
SSIII-2	Ap enzyme set i, Ap enzyme set iii, Am enzyme set i, Am enzyme set ii	Ap short and intermediate chains, Am short and intermediate–long chains	6–24; 28–58; 100–500; 500–1500
SSIV-1	Ap enzyme set i, Ap enzyme set iii	Ap short and intermediate chains	6–24; 28–58
SSIV-2	Ap enzyme set i	Ap short chains	6–24
SBEI	Ap enzyme set i, Am enzyme set ii	Ap short chains, Am intermediate–long chains	6–24; 500–1500
SBEIIa	Am enzyme set i, Am enzyme set ii	Am short and intermediate–long chains	100–500; 500–1500
SBEIIb	Am enzyme set i, Am enzyme set ii	Am short and intermediate–long chains	100–500; 500–1500
GBSSI	Ap enzyme sets i, iii and v, Am enzyme sets i and ii	Ap short, intermediate and long chains, Am short and long chains	6–24; 28–58; 68–78; 100–500; 500–1500

**Table 2:** Isoforms and functional properties correlated to starch fine structure.

DP Region [103]		Isoform [103]	Digestibility (Crops)	RVA Properties
DP 6~12	Ap short chains, Ap ES 1	SSI, SSII-2, SSII-3, SSIII-2, SSIV-1, SSIV-2, SBEI, GBSSI	RDS(+), SDS(–), RS(–) [113] (Rice, Maize)	BDV(+) [123,124,125]
DP 12~24			RS(+) [126] (Maize, Canna, Potato, Yam)	BDV(+) [123,124,125]
DP 24~40	Ap intermediate chains, Ap ES 2	SSII-1, SSII-2, SSII-3, SSIII-1, SSIII-2, SSIV-1, GBSSI	RDS(–) [113,127] (Rice, Maize)	Unknown
DP 40~100	Ap long chains, Ap ES 3	SSII-3, GBSSI	SDS(+) [115] (Rice, Maize)	BDV(–) [123,124,125]
DP 100~500	Am short chains, Am ES 1	SSI, SSII-3, SSIII-1, SSIII-2, SBEII, GBSSI	Digestion rate(–) [119,120,121] (Rice)	Unknown
DP 500~1000	Am intermediate chains, Am ES 2	SSI, SSIII-1, SSIII-2, SBEI, SBEII, GBSSI	Digestion rate(+) [122] (Rice)	RVA profile [41]
DP 1000~1500	Am long chains, Am ES 2		Unknown	Unknown

Notes: Ap, amylopectin; Am, amylose; ES, enzyme set; +, positive correlation with relative DP region; –, negative correlation with relative DP region.

## Data Availability

Data are available from the authors on request.
